# Advanced Practical Strategies to Enhance Table Egg Production

**DOI:** 10.1155/2022/1393392

**Published:** 2022-10-30

**Authors:** Karim El-Sabrout, Sarah Aggag, Birendra Mishra

**Affiliations:** ^1^Department of Poultry Production, Faculty of Agriculture (El-Shatby), Alexandria University, Alexandria, Egypt; ^2^Department of Genetics, Faculty of Agriculture (El-Shatby), Alexandria University, Alexandria, Egypt; ^3^Department of Human Nutrition Food and Animal Sciences, University of Hawaii at Manoa, 1955 East-West Road, Honolulu, HI, 96822, USA

## Abstract

The global demand for table eggs has increased exponentially due to the growing human population. To meet this demand, major advances in hen genetics, nutrition, and husbandry procedures are required. Developing cost-effective and practically applicable strategies to improve egg production and quality is necessary for the development of egg industry worldwide. Consumers have shown a strong desire regarding the improvement of hens' welfare and egg quality. They also become interested in functional and designer foods. Modifications in the nutritional composition of laying hen diets significantly impact egg nutritional composition and quality preservation. According to previous scientific research, enriched egg products can benefit human health. However, producers are facing a serious challenge in optimizing breeding, housing, and dietary strategies to ensure hen health and high product quality. This review discussed several practical strategies to increase egg production, quality, and hens' welfare. These practical strategies can potentially be used in layer farms for sustainable egg production. One of these strategies is the transition from conventional to enriched or cage-free production systems, thereby improving bird behavior and welfare. In addition, widely use of plant/herbal substances as dietary supplements in layers' diets positively impacts hens' physiological, productive, reproductive, and immunological performances.

## 1. Introduction

**Figure 1:**
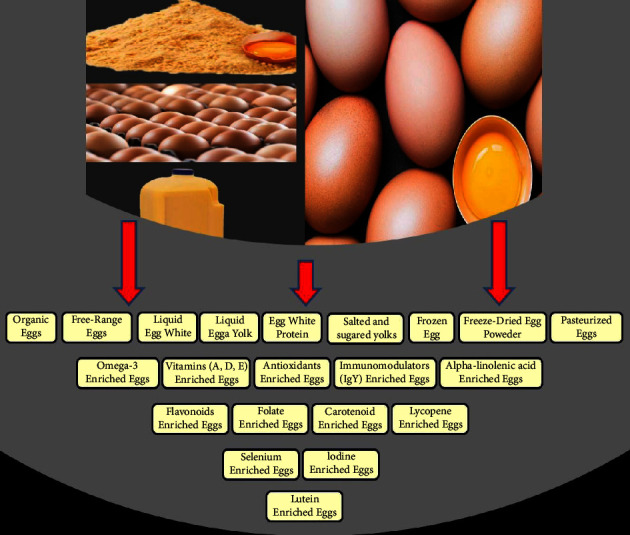
Unconventional and designer egg products.

The poultry industry is among the fastest-growing agricultural industries worldwide. It provides large quantities of high-quality protein (meat and egg) for human consumption. Recently, poultry industries faced many challenges including global population growth, climate change, feedstuff shortage, economic recession, and the emergence of diseases. To confront these challenges, there is a critical need to enhance productivity and improve poultry product quality. The egg industry is one of the main poultry industries that attracts many investments across the world. In several countries, eggs are considered as the cheapest and healthiest source of animal protein. The egg is a natural product (a nutraceutical = a combination of nutrition and pharmaceutical) that provides essential nutrients for human health. A complete chicken egg contains high level of water (approximately 75%) and comprises organic and inorganic components [1, 2]. Eggs are rich in amino acids, fatty acids, minerals, and vitamins; thus, they are considered a complete food and occupies a special place among consumers. Natural eggs and egg-derived products contain antioxidant, anti-inflammatory, immunomodulatory, and anticancer components, which can help in the maintenance of biological processes in the human body and protect organs such as heart from many diseases [3–6]. They are also referred to as functional food [7, 8] due to their beneficial effects on human health along with their basic nutritional impact. Unconventional egg products, such as liquid egg white, egg white protein, frozen egg, freeze-dried egg powder, pasteurized eggs, and homogenized eggs, have become popular among consumers worldwide (Figure 1). Despite the egg's high nutritive value, some nutritionists recommend limiting egg consumption to reduce cardiovascular diseases. Numerous clinical and epidemiological studies have found no evidence of a link between egg-derived cholesterol consumption and an increase in blood total cholesterol [9].

## 2. Current Egg Production and Challenges

Laying hens have a high potential to convert beneficial nutrients received from diets to eggs. They can also produce more antibodies than other livestock, considerably reducing the number of animals required to produce antibodies [10–12]. Poultry eggs can be enriched by adding certain vital components (active biological substances) that are not normally found in the product, providing health benefits and high nutritional value. They are consumed as part of a normal diet and at the same time they have the potential to enhance immunity and reduce the risk of diseases. Consequently, breeding and nutritional programs for layer farms have been developed to produce egg products with additional beneficial substances, such as omega-3, and a reduction in hazardous substances, such as low-density lipoprotein cholesterol (changing the fatty acid profile). Eggs could be designed to be high in vitamins, minerals, choline, polyunsaturated fatty acids such as omega-3, antibodies such as IgY, and antioxidants such as lutein. Designer foods are ordinary foods that have been fortified with health-promoting substances. These foods have the appearance of regular foods and are consumed on a regular basis as part of the diet. The designer eggs are produced with a high concentration of health-promoting components, such as omega-3 fatty acids, vitamin E, antioxidants, carotenoid pigments, and minerals such as zinc, in addition to reducing yolk cholesterol levels (Figure 1). Although, the developed designer-egg products are interesting and appealing to consumers [13, 14], they should be subjected to legal, ethical, social implications, and economic studies before they can be widely accepted.

**Figure 2:**
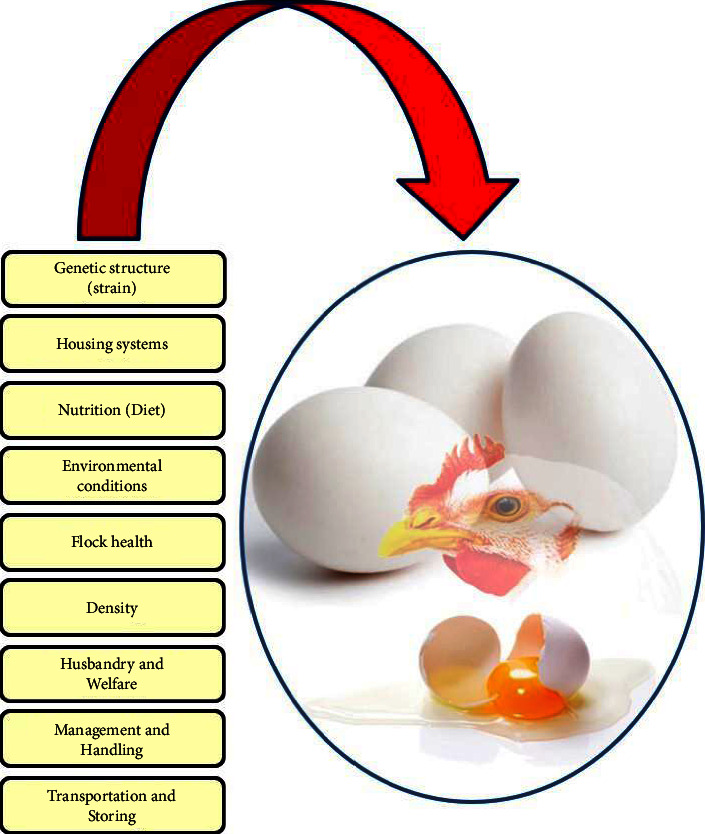
Schematic overview of the main factors that can affect egg production and quality.

The rate and quality of egg production are major determinants of layer farms' economy and are affected by several genetic and environmental factors (Figure 2). The poultry housing system and nutrition are important environmental factors that can improve animal welfare and provide higher-quality products [15]. Improving hen health and producing high-quality and safe eggs are now priorities for all laying farms. Organic eggs are produced from free-range farms that adhere to various management, husbandry, and egg production standards, including diet component source, stocking density, temperature, lighting, ventilation, flock health, and a biosecurity system. Organic production refers not only to the final product's quality, but also to the entire production process, which must be subject to strict quality and security controls. In the near future, the production stage of layers will be routinely extended to improve production economics and environmental footprint. Several layer farms are already producing up to 500 eggs per hen throughout their production cycles, reducing the need for replacement layers and improving economics and sustainability. It will be challenging for breeders and researchers to optimize genetics and production systems to keep these hens healthy. On the other hand, breeders' and consumers' awareness of poultry welfare has grown in recent years, and as a result, consideration for higher-quality egg production has increased. Consumers, particularly in developed countries, have become acutely aware of the relationship between food and health. They are more interested in the diet components that birds consume and their quality. In addition, they are interested in poultry products derived from alternative and enriched housing systems [16, 17], which are natural, organic, and safer for human health. Due to their beneficial effects, many of these egg products are available in the United States, China, and certain European countries and are widely accepted by consumers. Recently developed egg products are an obvious example of these healthy products that are also economically interesting because it allows poultry producers to introduce new products in a rewarding exchange. Thus, consumers are willing to pay a premium price for many enriched products, which are 15–20% higher than the average product price, as stated by Siró et al. [18].

Currently, commercial laying hens start laying at around 20 weeks of age and continue to lay for 52 weeks. Scientists and egg producers have developed practical breeding, husbandry, and nutrition strategies to improve hen egg production, achieve the highest quality products, and extend the production period (Table 1). They have demonstrated that by adhering to certain breeding and management programs it is possible to keep our long-life layers up to the age of 100 weeks and maintain egg production quality [40]. Keeping laying flocks for longer production periods has critical advantages for animal welfare, economics, and enhances sustainability. It reduces production costs by reducing downtimes, and the pullet/once-per-cycle costs can be spread out over a much longer production period. Therefore, this review aims to discuss these practical strategies and offers specific recommendations to breeders and producers to increase their laying flock egg production and produce healthy food.

## 3. Breeding and Genetic Improvement Strategy

Egg production is considered one of the most important economic traits in the poultry industry; thus, several breeding researchers and geneticists occupied improving this trait as the primary target in layers breeding programs. Egg production can be measured using various methods, including hen-day egg production, hen-housed egg production, and egg mass. In modern poultry breeding, egg number is the most important desirable trait in selecting hens with a higher egg-laying capacity [19]. It is well recognized that the egg production traits are controlled by multiple complex genes [41]. The molecular marker-assisted selection technology can be an effective, accurate, and rapid method to improve various egg production traits. It has been widely used in animal breeding due to its numerous advantages over conventional breeding [42, 43]. In addition, the results of molecular genetic studies are crucial in breeding value prediction systems and the development of commercial strains and lines. Association studies of identified single nucleotide polymorphisms (SNPs) in the genes correlated with egg production traits have resulted in an understanding of the normal differences between individuals, which will be useful for improving animal breeding with potential economic benefits [44]. The investigation approach for a specific desirable SNP entails a novel and lengthy process of identifying the DNA molecular marker for a major effect gene. Holsinger and Weir [45] emphasized the significance of discovering various SNPs in the genomes of many species, allowing for the exploration of genome-wide signatures in selection through an assessment of variation in marker allele frequencies among these populations. SNPs of several candidate genes were used to identify genes associated with productive traits [46, 47]. The poultry industry has highly valued selection programs based on egg production candidates, such as reproductive hormone candidates [48]. Liu et al. [19] investigated egg production's genetic architecture and measured several traits such as hen's age at the first egg. Using a linear mixed model, they performed genome-wide association studies (GWAS) on 1078 Rhode Island Red hens. Based on marker-assisted breeding selection, their findings could provide promising genes and SNP markers to increase egg production. Based on the importance of the hen's ovary in egg production, numerous studies have been conducted to identify potential candidate genes at the transcriptome level and their spatiotemporal expression profiles at various physiological statuses of hens or egg-laying conditions, as well as SNPs of genes associated with egg production [20]. Molecular marker genotyping is a common requirement for Quantitative Trait Loci (QTL) mapping and the GWAS approach, and it can be the basis for combining these approaches. The prospects for broader selection indices based on molecular genetics are expanding, and producers are already utilizing these advanced tools to resolve some of the egg industry's major issues for the benefit of the hens, the consumer, and the environment.

As biotechnology advances, food biofortification using technologies such as recombinant DNA and fermentation procedures has gained attraction in the poultry sector. Novel biotechnology aids in developing genetically modified chickens (transgenic chickens), which could be used to produce eggs with medicinal properties such as insulin for diabetes treatment and various antibodies for microbial toxins treatment [22]. Transgenic layers could be used to generate antibodies in their egg's albumin [8]. Egg yolk antibodies (EYA) are antigen-specific antibodies produced by injecting a specific antigen subcutaneously or intramuscularly into hens, resulting in the development of IgY antibodies [49, 50]. Eggs could be used as a source of immunogens that enhance human's immune system, particularly children, and protect them against several diseases [23]. EYA can also be used as antibacterial and antiviral agents in humans and animals to treat enteric infections caused by *E. coli*, coronaviruses (such as COVID-19), and rotaviruses [51].

The poultry industry's successful breeding and genetics progress has narrowed the gap between availability and animal protein demand by supplying meat and eggs [52]. Modern breeding methods such as genomics, fully automated measurements, and other massive investment in expanding our breeding research and development programs have contributed significantly to the success of the sector of laying hens. Breeding efforts are ongoing to improve layer's feed conversion to produce more egg mass per gram of feed consumed. Improving laying persistency has a significant impact on the birds' feed efficiency. Birds that keep healthy and productive have a lower risk of becoming fat and can use the nutrients in their diets much more efficiently than birds that stop producing or go longer between egg productions. Lately, the main reason for flock depletion is not often related to egg production persistence, but to eggshell quality that deteriorates with age. Poor quality eggs, particularly those with weak shells, can raise egg packing costs and cause customer complaints [53]. Eggshell is the outermost calcified covering composed of approximately 95% calcium carbonate crystals. It protects eggs from microbial invasion and physical damage and preserves egg quality. Almost 10% of eggs produced by laying farms are broken due to cracking of weak eggshells, causing significant financial loss to the egg industry. Breeders and researchers have been constantly developing strains of long-life hens to achieve higher egg yield per hen [54], and some improvements in eggshell strength have been achieved. The genetic regulation of eggshell formation and its biological pathways are extremely complicated and not fully understood. Sah et al. [21] identified novel genes and biological pathways involved in eggshell biomineralization, nevertheless, more research is required to understand better the eggshell genetic structure and the genetic factors that affect the eggshell quality.

Scientists and egg producers have recently increased their focus on selection to improve egg quality. Successfully keeping laying hens productive for 100 weeks is no longer an exception. Several egg producers worldwide manage to express their chickens' full genetic potential, but the early depletion wastes the genetic potential of today's laying hens [53]. Developing long-life laying hens can help breeders save production costs, improve chicken welfare, and reduce environmental carbon footprint [53, 54]. A long-life laying hen is capable of producing 500 eggs in a 100-week laying cycle. Improving hen nutrition and carefully monitoring her behavior to provide appropriate welfare are required to fully realize the benefits of genetic selection for improved lay persistency and egg quality stability [54]. However, egg production is a complex metric trait, and many authors are interested in studying egg production and related traits such as age and body weight at sexual maturity. The key to achieving genetic improvement through the value chain is balanced breeding for all productive traits [53]. Bird's health and welfare are key factors that should be included in breeding programs. To keep up with the longer cycles required, these factors should be provided to today's commercial laying hens. This balanced trait approach necessitates extensive knowledge and consideration of the bird's physiology and nutritional needs during the production stages. It also depends on the management practices and the reproductive status of the birds. Long-life laying hens can only be successfully kept for longer cycles if they are properly handled. Breeding scientists apply additional selection pressure to the pullet's development, robustness, and livability through recent breeding programs [53]. Robustness is a broad term that often refers to a laying hen's ability to deal with challenges such as diseases or environmental stressors. It begins as early as the age of a day old. It is critical that laying hens can deal with illness and other stressors. New techniques are being developed to genetically modify birds to make them resistant to a specific disease [53].

Despite significant progress in chicken breeding programs over the last 50 years and the use of modern biotechnological approaches, some traditional breeding tools continue to hold a special place and importance among researchers and breeders in developed and developing countries. Crossbreeding is one of these tools, which is still used scientifically and commercially in many countries such as China, India, Turkey, Pakistan, and Egypt. It is preferred for various reasons, including its effectiveness, ability to serve multiple purposes, ease of implementation, lack of the need for more advanced methods or materials, and low cost. Crossbreeding (genetic crossing) plays a significant role in increasing chicken productivity. Furthermore, it increases the heterozygosis of nonadditive genes, resulting in heterosis, which is critical for resistance to adverse environmental conditions. Several countries are suffering from a scarcity of high-yielding commercial chicken lines that are also adaptable. The indigenous chickens' genetic resource base can serve as a foundation for genetic diversification and improvement, resulting in new strains better adapted to local conditions. Indigenous chickens have desirable characteristics such as disease resistance, greater robustness against stressors, superior adaptability to local climatic conditions, higher survival than commercial hybrid strains under local production conditions, and excellent product flavor and taste [55–58]. However, breeders use two tools to improve egg production in poultry: the first is to select the best birds to be used as the next generation's parents [59] and the second is crossbreeding programs. Moreover, analyzing the differences in productive performances of crossbreed aids in identifying the best possible hybrid vigor combinations based on the target objectives [60]. Previous studies have shown that crossing improves genetically quantitative characteristics such as bird body weight and egg production [57, 61–63]. Crossings between adapted local chicken and exotic standard breeds would allow exploiting the rusticity of the first and the productive performance of the latter to produce adapted and more productive genetic types; in addition, hybrid vigor (heterosis) can be a useful tool to generate several chicken strains [64, 65]. The synthesis of the new hybrid line is essential for achieving high laying hen productivity. This new hybrid line represents the extent to which a crossbreed's performance in one or more traits is better than the average performance of the two parents.

Sharma et al. [66] concluded that laying hen strain greatly impacts performance, egg quality traits, and cloacal and eggshell microbiology. Hy-Line Brown hens outperformed W-36 in egg production and quality parameters in enriched colony cages. Thus, they reported that the laying hen strain should be considered when selecting a housing environment. Therefore, breeders and researchers are now focusing on the interaction of genetics and the environment to reach birds' full genetic potential. It is critical to fully comprehend this relationship. It will not be enough to improve hens' genetic structure and neglect the environmental conditions surrounding them.

## 4. Poultry Housing Enrichment Strategy

Better management and welfare, as well as the provision of a suitable environment, are required prerequisites for exploiting genetic potential in conjunction with genetic improvement [67]. Recently, there has been a strong consumer desire for improved animal management and welfare in poultry farms, particularly in egg production. Scientists and breeders face a difficult challenge in determining the ideal environmental conditions for animals considering existing environmental changes [68]. Furthermore, the egg industry's sustainability should be investigated further to improve housing systems that meet the needs of laying hens and minimize the ecological footprint of egg production [40]. Poultry housing is one of the most important environmental factors affecting poultry rearing, health, welfare, and production. The housing system includes several critical factors, such as stocking density, which can greatly impact bird performance, product quality, and welfare, either directly or indirectly. The introduction of animal welfare considerations and perceptions has resulted in additional changes in layer hen housing, influencing production practices in the commercial egg industry [69]. Developing housing systems for layers, such as enriched cages or free-range systems, has resulted in advancements, with welfare concerns driving this progress [24]. Konkol et al. [25] found that providing additional feeders in furnished cage systems improves laying hen behavior and welfare. Consumers have shown a particular interest in poultry products derived from alternative housing and free-range systems because these products are considered as fresh, clean, healthy, and contain fewer substances that can harm human health [16, 17, 28]. Due to increased awareness of animal welfare and environmental footprint among breeders and consumers, many egg production farms are converted into enriched cages, cage-free, and monitoring systems. In some European countries, decisions such as changing the egg production system in poultry farms should be subjected to a sustainability assessment that includes animal welfare, environmental, social, and economic dimensions. Environmental control strategies are alternatives for reducing the environmental impact of cage-free and enriching aviary housing systems. Assessing the consequences of these strategies has become an important procedure for developing optimal laying hen production systems with a low environmental impact and great profitability [70].

Traditional conventional battery cages have long been regarded as the most proficient and efficient for egg production farms, but the cage system negatively impacts chicken welfare [71]. The concept of animal welfare has been transformed into specific guidelines; consequently, alternative production systems are becoming more widespread for the better fulfillment of welfare aspects in developing countries [72]. Aviaries and enriched cage systems are two broad categories of alternative production systems, including environmental enrichment and cage furnishing. Environmental enrichment or cage-furnishing is the modification of cages or enclosures with tools to improve birds' biological functions and behavioral activities [73, 74]. Environmental enriched cages are designed with specific features that encourage birds' daily activities and provide more opportunities for better space utilization [29]. Sufficient space in cages and aviaries is preferable for birds to express natural behaviors, such as wing-flapping, perching, nesting, dust bathing, walking, and exercise [28, 30]. Moreover, birds exhibit better leg health and decreased fearfulness as an effect of the environmental enrichment. An increased available space and greater freedom of movement lead to increased physical activities, which positively impact product quality [28]. Improved welfare and behavior for laying hens in enriched cages and aviaries enrich comfort levels over conventional cages [26, 75]. Compared to enriched cages, conventional cages may result in more walking time, behavioral transitions, higher posture, and increased stress level, whereas furnished cages promote more preening [71]. Enriched cages are beneficial in terms of stress, fear responses, and aggression [76, 77]. Due to their even distribution in cages, hens' productive and reproductive performance improves in enriched cages [29].

It is well known that table eggs can be produced on deep litter, in cages, and in free-range systems. For several years, there has been a consistent decrease in the number of hens housed in conventional cage systems in favor of enriched or noncaged systems (such as free-range and organic systems) [78, 79]. However, compared to traditional cages and the deep-litter system, the enriched cages, the free-range, and organic systems promoted a higher proportion of comfort behaviors, ensuring a greater level of behavioral welfare for laying hens [78, 79]. Aviaries, enriched cages, and free-range systems are supposed to help the birds in terms of housing and natural behavior, allowing them to increase overall bird activity, reduce stress, lower the risk of immunosuppression, and enhance their health status and welfare [78, 80].

On the other hand, alternative production systems are still not commercially used in several developing countries worldwide. According to Sharma et al. [66], laying hen housing has a considerable impact on performance, egg quality measures, and cloacal as well as eggshell microbes. Layers housed in conventional cages and free-range had higher egg production and lower feed conversion ratio than hens housed in enriched colony cages. However, most of the measured egg quality parameters for free-range were intermediate between conventional cages and enriched colony cages housing environments. In addition, free-range chickens had higher eggshell bacterial contamination than traditional cages and enriched colony cages. In agreement, Leyendecker et al. [81] reported that the percentage of dirty eggs was higher in free-range production systems than in indoor cage systems. Although the free-range systems are beneficial for comfort bone and feather traits, they also have disadvantages, such as increased feed intake, dirty eggs, and increased leg deformities and foot problems [78]. However, the production traits (such as the final body weight), feather quality, and foot infections of laying hens in conventional and enriched cages were comparable, according to Dikmen et al. [78]. On the contrary, Appleby et al. [82] reported reduced foot problems and feather damage in enriched cages than in conventional cages, depending on the number of birds per cage. Moreover, aviary systems cause more dirty plumage, wounds, keel abnormalities, and foot deformities than enriched and conventional cages. Overall, feather cover and feather loss patterns were caused by head pecking in the aviary system and cage abrasions in enriched and conventional cages [83]. Englmaierová et al. [84] discovered that traditional cages had the highest egg production, lowest daily feed consumption, and lowest feed conversion ratio when compared to litter and aviaries. In addition, eggshell and albumen quality were better in conventional cages, while hens kept in enriched cages and aviaries lay eggs with a higher yolk index. The housing system had a substantial impact on the overall number of bacteria on the egg surface as well as the microbiological contamination with *Enterococcus* and *Escherichia coli*. The highest values for total bacterial contamination were found in eggs laid on litter, while the lowest values were found in eggs from conventional and enriched cages. Authors concluded that enriched cages and aviaries are a better alternative for conventional cages than litter in terms of egg safety. Furthermore, Usman et al. [27] reported that hens reared in enriched cages showed higher production performance and egg quality than those raised in conventional cages and aviary systems, while hens raised in the aviary system had a higher fertility rate.

Several housing and husbandry systems are currently in commercial use worldwide. These systems are developed by each country based on its priorities and economy. Poultry housing systems, in general, have advantages and disadvantages that vary according to the line (strain) used, local surrounding environmental conditions, and farm management. Nevertheless, it remains a fixed and agreed-upon rule that birds raised on the litter (floor) or in outdoor systems are more susceptible to infection than those raised in enriched cages or indoor systems. In addition, during pullet rearing and production, enrichment layers' housing (nest boxes and perches) improves hens' behavior, adaptation, welfare, and product quality.

## 5. Dietary Strategy for Egg Enrichment and Egg Production Improvement

The egg is nature's most complete food, with high-quality proteins and a 2 : 1 unsaturated fat to saturated fat ratio. It is an excellent source of iron, phosphorus, other minerals, and vitamins except for vitamin C [85]. High-quality protein foods like eggs can normally support infants, children, and adolescents' growth and development. A large egg (65 g average weight) contains approximately 200 mg of cholesterol. Dietary interventions have received the most attention in research to lower egg cholesterol. Supplementing natural substances such as garlic, plant sterols, and n-3 polyunsaturated fatty acids in poultry diets reduces yolk cholesterol levels [86]. In addition, eggs are a natural source of high choline concentrations, with approximately 147 mg per large egg [87, 88]. Choline is a crucial nutrient involved in many biological activities, and it regulates growth and development in infants and toddlers through multiple biological pathways. Choline is also important for cell membrane integrity, methyl metabolism, cholinergic neurotransmission, transmembrane signaling, lipid, and cholesterol transport and metabolism [87–90]. On the other hand, eggs are deficient in vitamin C (ascorbic acid). Since animals cannot synthesize ascorbic acid, this vitamin should be obtained through the diet, particularly from vegetables and fruits. Ascorbic acid is a powerful antioxidant that plays an important function in body metabolism and immunological regulation [91].

As a result of their unhealthy habits, many people suffer from malnutrition and a lack of several essential nutrients in their diets. Recently, consumers paid attention to the nutritional enrichment of laying hen diets for bird health, performance, and even egg quality. Enriched eggs (also known as modified eggs) differ from traditional eggs in terms of certain components, characteristics, and effects. They are commonly referred to as designer eggs because they provide consumers with various nutritional and health benefits. The enrichment of widely consumed foods has been proposed as a strategy to increase omega-3 fatty acids (n-3 FA) consumption. Omega-3 fatty acids are polyunsaturated fatty acids (PUFA), also known as n-3 fatty acids. Due to eggs' widespread consumption and the ability to change their omega-3 content through hens' diets, they are considered an ideal target for enrichment [92]. The n-3 FA, also *α*-linolenic acid, is considered an essential nutritional element because it cannot be produced by the human body and must be obtained through diet. As birds and animals cannot synthesize these n-3 fatty acids, they are required in the diet. Fish oil, linseed/flaxseed oils, rapeseed/canola oils, sunflower oil, soyabean oil, and marine algae are good sources of omega-3 fatty acids. Unfortunately, foods high in these n-3 fatty acids, such as linseed and fish oils, have an unpleasant flavor and consistency, making them unappealing to most people. Therefore, it is very important to increase the level of these acids in the egg without affecting their sensory attributes, whereas by consuming one egg daily, human requirements are met [93]. Omega-3 fatty acids have been found to improve blood vessels and the heart, decrease atherosclerosis risk, reduce inflammatory processes, and have anticarcinogenic effects in some cases [94–96]. According to Ahmad et al. [31], feeding laying hens high PUFA with a lower linoleic acid to the *α*-linolenic acid ratio diets can result in PUFA-enriched eggs with negligible adverse effects on the production performance and egg composition. Increased PUFA content in eggs can make them more susceptible to oxidation and flavor change [97, 98]. According to Surai and Sparks [98], the solution is in the simultaneous addition of antioxidants—vitamin E, selenium, and carotenoid, which increases polyunsaturated fatty acids stability during eggs storage and cooking, improves flavor, and egg is additionally enriched with antioxidants that have positive physiological benefits for consumers. Commonly used for this purpose are tocopherol (vitamin E), selenium, and carotenoids. Vitamin E and carotenoids can be added in a wide range because they have no negative effects on egg quality or consumer health [93]. According to Narahari [99], the vitamin E content of enriched eggs can be increased up to fourfold, and in the case of carotenoids, the egg yolk color must be considered. Several consumers associate egg yolk color (light-yellow to orange) with hen health and egg quality [100]. The addition of selenium as an antioxidant must be carefully dosed because higher concentrations of this element harm human health [101].

Carotenoids (lipid-soluble bioactive compounds)-enriched eggs are a relatively recent egg product feature. Carotenoids are naturally present in egg yolk in varying amounts depending on the hen's diet. In general, hens with deep yellow or orange yolks are preferred over those with pale yolks. According to Sünder et al. [32], adding marigold flower meal (*Tagetes erectus*) and spinach (*Spinacia oleracea*), a mixture of 25% marigold flower and 75% spinach (as carotenoid sources), is appropriate for improving yolk color in organically produced table eggs. Lutein, zeaxanthin, and capsanthin (a natural red dye of the xanthophyll) are carotenoid pigments rich in provitamin A. They can serve as antioxidants and anticarcinogenic agents and provide attractive yolk colors. Feed fortification with natural sources, such as marigold and alfalfa extracts, are sources of lutein. Lutein, as an antioxidant, serves several important functions in the body, including protecting the retina [86]. Other sources such as corn and red pepper (chili pepper) contain various carotenoids, including zeaxanthin and capsanthin, which are also important for eye health [102]. Furthermore, lycopene (a hydrocarbon carotenoid) has been shown to have powerful antioxidant properties that can help reduce the risk of prostate cancer [103, 104]. Egg lycopene enrichment can be achieved by fortifying feed with tomato powder (5–10 g/kg diet) [33]. This supplementation increased egg production and improved egg yolk color. In addition, lycopene can potentially reduce yolk lipid peroxidation [33].

Several authors ensured that functional food could reduce the risk of disease incidence and enhance human health [18, 105–107]. Enrichment of eggs with immunomodulators is considered a recent egg product aspect. The egg is a viable alternative source of antibodies. Immunoglobulin Y (abbreviated as IgY) is the most common immunoglobulin in egg yolk. Chicken eggs are rich in antibodies such as IgY, which is less expensive and more effective than mammalian immunoglobulin IgG [108]. This IgY can treat human rotavirus, *Staphylococcus*, *Streptococcus*, and *Salmonella* infections [108]. Dietary manipulations can increase the level of IgY in the egg. The egg's IgY level can be raised using certain herbal dietary supplements high in omega-3 fatty acids and antioxidants. Basil leaves and other herbs, such as rosemary, turmeric, garlic, fenugreek, and spirulina, can improve the IgY level in the egg [86, 108].

Furthermore, the importance of increasing folate consumption by humans is becoming more widely recognized. Folate refers to a group of water-soluble molecules based on the structure of folic acid, or pteroylmonoglutamate, but varies in oxidation state and glutamate residue count [109]. Women's increased periconceptional consumption of this vitamin has been proven to lessen the occurrence [110] and recurrence [111] of neural tube abnormalities in children, such as spina bifida. Foods do not naturally contain folic acid in significant proportions. It is, however, the form employed in vitamin supplements, fortified foods, and vitamin premixes due to its stability and commercial availability. The effectiveness of these methods in increasing serum folate levels in the general population has yet to be completely investigated [112]. However, additional efforts, such as using supplements in specific target groups (women of child-bearing age) and training consumers to eat folate-rich foods, may be required to ensure that all population segments get enough folate. Natural folate levels in eggs are around 22 *μ*g per large egg [113], equivalent to 6% of the newly established adult daily folate requirements [114]. Increasing egg folate content can promote consumer acceptability of eggs as a portion of healthy food [115].

Flavonoids are dietary polyphenolic chemicals found in plants, such as quercetin, and they have an important role in improving birds' productive performance and biological features such as phytoestrogenic, immune, antimicrobial, antioxidant, and anti-inflammatory effects [116–118]. Vitamin E (also known as tocopherol or alpha-tocopherol) has been shown to have egg-producing and positive immunoregulatory effects. According to Amevor et al. [34], supplementing the diet with quercetin and vitamin E increased the laying rate (84.5%), enhanced immunity response (IgM and IgA concentrations), and improved the yolk weight, yolk height, and Haugh unit in the aging breeder hens compared with those fed a basal diet. Therefore, they concluded that combining quercetin and vitamin E positively affects egg production, egg quality, and immunity of aging hens. Due to flavonoids' beneficial effects, egg producers can enrich chicken eggs in diets to provide a vital natural source of flavonoids and promote consumer health.

Several plants contain high levels of omega-3 fatty acids, carotenoids, folic acid, flavonoids, and other biological components. They are preferable to be used in layer hens' nutrition for many purposes, including increased egg productivity and quality and achieving high economic profit. Rather than antibiotics, new natural materials from plant sources have been used as feed additives in poultry diets in recent years. These materials must provide safe and high-quality food [68, 119]. Some of them are frequently used in poultry diets as commercial additions and alternative feed supplements to improve animal productivity and welfare due to their multiple growth and health benefits [120–122]. They also have the potential to improve the balance of the intestinal microbiota, which is important in regulating metabolism, intestinal epithelial proliferation, and vitamin synthesis [123, 124]. These natural additives are now being developed as nano-substances to treat several diseases in poultry farms, including nutritional deficiency (malnutrition) and coccidiosis. They can also stimulate various gene expressions related to growth, metabolism, and immunity [125].

Current poultry breeds are selected for higher meat and egg production. They are metabolically more active, resulting in the generation of cellular free radicals. These free radicals disrupt cellular processes by escalating oxidative stress, inflammation, and immunosuppression. Researchers have recently examined several botanical products and their bioactive compounds for their ability to scavenge free radicals and restore cellular activities necessary for poultry health and productivity. Botanical components (such as plant medicinal seeds and plant essential oils) are gradually gaining popularity in poultry farms due to their nutritional value and therapeutic properties, as well as leaving no residue in products [15]. Plants' essential oils, for example, have been routinely used in chicken diets to keep birds healthy and improve their productive performance. These essential oils include active components that positively impact physiological functions and medicinal features, such as anti-inflammatory and antibacterial effects [126, 127]. They contain phytobiotics, phenols, flavonoids, tannins, and essential oils, which have a multitude of roles in the birds. In addition, botanical or herbal medicine products are considered phytogenic feed additives for birds, improving egg weight, ovary characteristics, and decreasing yolk trimethylamine levels in laying hens [128]. Thyme (*Thymus vulgaris* L.) is considered one of the most common medicinal plants worldwide. The essential oil in dry thyme (active extract) is a combination of monoterpenes, primarily thymol. Thyme also contains phenolics, some biphenyls, and flavonoids which have been shown to have antioxidative properties and other benefits for birds [129–132]. Thyme extracts are advised in laying farms to improve egg quality, especially the fatty acid profile in the yolk [133]. Egg lipid profiles can be altered by feeding hens plant seed oils (such as flaxseed and rapeseed) to reduce n-6 fatty acid content while increasing n-3 fatty acid content [35, 36]. Yalçn et al. [134] revealed that layers' diet enriched with dry thyme leaves at 2% has several positive impacts on laying hens' physiological and productive performance and their antioxidant activity. Thyme leaves significantly influenced egg production and quality and its nutritive value by decreasing bad yolk cholesterol (low-density lipoprotein (LDL)) and total saturated fatty acid concentrations and increasing n-3 FA concentrations. It also raises the *α*-linolenic acid level of eggs while significantly reducing palmitic acid content in the yolk. Furthermore, dietary thyme at 2% can reduce yolk malondialdehyde, blood unhealthy cholesterol, and triglyceride levels. Dietary supplementation of thyme can enhance poultry egg production and the humoral immune response without harming the birds [133–137]. The precise mechanism of action could be related to intramuscular fat stimulation and flavor amino deposition. According to Hashemipour et al. [138], phytogenic compounds containing thymol and carvacrol increased digestive enzyme activity, antioxidant capacity, and slowed lipid oxidation in birds. Furthermore, thyme contains flavonoids, which can increase the activity of vitamin C, which acts as an antioxidant, and thus improve immune function [138, 139]. It also plays an important role in reducing stress on birds, particularly laying birds and especially during the hot seasons [37].

Imran et al. [38] reported that extruded flaxseed meal up to 20% could be supplemented in hen diets to maximize *α*-linolenic acid and docosahexaenoic acid deposition in egg yolk. Lee et al. [140] investigated the fecal microbiota composition and egg quality traits of laying hens fed alpha-linolenic acid-rich flaxseed oil (0.5–1%) for 8 weeks. They concluded that diets high in alpha-linolenic acid positively influenced the egg and fecal microbiota in laying hens. Working on turmeric's (*Curcuma longa* L.) major component “Curcumin,” Sharma et al. [141] reported that curcumin had been identified for various biological activities in the animal's body. It can reduce free radicals and improve antioxidative responses [142, 143]. Thus, it becomes a vital medical and antioxidant substance in poultry diets, especially in tropical areas where high temperatures throughout the year can delay growth, decrease egg production, and increase disease outbreaks and mortality of birds [144]. It can improve feed intake and significantly lower cholesterol in poultry products when added to poultry diets at a 1–5% concentration. Eggs' cholesterol was reduced by 16 and 25% when layers were fed 1 and 4% turmeric in the diet, respectively [145]. Compared to a control group, birds fed a high carbohydrate content supplemented with turmeric for a month before sexual maturity produced more eggs (20%). The active turmeric extract (curcumin) influences lipid metabolism and inhibits peroxidation [146]. It stimulates bile production, which is necessary for lipid emulsification [147].

Fenugreek seeds (*Trigonella foenum graecum*) are known for their medicinal properties, including antibacterial and anti-inflammatory effects [148, 149]. Galactomannan is the main polysaccharide in fenugreek seeds, representing approximately 50% of the seed weight [150]. Samani et al. [151] worked on laying chickens and concluded that using 1% fenugreek can improve bird feed intake and egg yolk color, particularly in the second production cycle. Fenugreek supplementation at 0.4% in laying diets improved egg production and quality [152]. Another example of a dietary strategy for improving egg production is ginger roots (*Zingiber officinale*), which are widely used as a medicine plant worldwide [153]. They contain vital volatile oils and other active substances, such as gingerols and zingerone, enhancing beneficial organisms and antioxidative activities in birds [154]. Zhao et al. [39] indicated that dietary supplementation of ginger powder (10–15 g/kg) in laying diets improved laying performance and serum antioxidant status of hens. In addition, oral administration of 100 to 150 *μ*l/kg body weight of ginger essential oil to laying Japanese quails increased egg weight and reduced egg cholesterol [155].

Pumpkin (*Cucurbita maxima*) and garden cress (*Lepidium sativum*) seeds are also nutritional and medicinal plant seeds that have recently piqued the interest of many researchers and breeders worldwide [156]. They contain essential aromatic oils making them excellent sources of unsaturated fatty acids, in addition to their high content of saturated fatty acids [157, 158]. They can also provide two types of omega-3: eicosapentaenoic acid and docosahexaenoic acid. They contain *α*-linolenic acid, which can be transformed into eicosapentaenoic acid and docosahexaenoic acid in the body [159]. In addition, pumpkin and garden cress seed oils contain a high concentration of vitamin E, zinc, and L-tryptophan [160–165], which can improve bird's productivity, immunity, and physiological functions, including antioxidant [166]. Martínez et al. [167] reported that adding 10% pumpkin to layer diet increases ether extract concentration and healthy fatty acids while decreasing total cholesterol and harmful fatty acids in the eggs. On the other hand, tocopherol (as a natural antioxidant), carotenoid (as a natural pigment), oleic acid (one of the main components of egg yolk flavor), and linolenic acid are found in garden cress oil and inhibit a variety of radicals [157, 168].

Chamomile flower (*Matricaria* sp.) is an additional herbal product that improves egg production. El-Galil et al. [169] reported that chamomile addition at 0.50 g/kg of quail diets can improve productivity, reproductive performance, and economical efficiency of laying Japanese quail. Furthermore, including 2.5–5 g chamomile/kg in a Japanese quail diet reduces the behavior of aggressive pecking and improves birds' welfare [170]. Chamomile contains flavones (such as apigenin), coumarins, essential volatile oils (1-2%), and chamazulene (10%) [171], which have antioxidant, anti-inflammatory, antimicrobial, and cholesterol-lowering effects [169–173]. Azolla (*Azolla pinnata*), a further example of a dietary strategy for egg production improvement, is a small aquatic fern plant that lives afloat on the water's surface and can resist hot temperatures. It is used as a feedstuff in poultry and livestock diets due to its high nutrient content. It can be used as a low-cost animal feed and a partial replacer for high-priced traditional proteins in poultry diets. According to Mishra et al. [174] and Al-Rekabi et al. [175], Azolla can be used in poultry diets at 25–30% for chickens and a higher rate of 40–45% for ducks and geese. The high enzyme concentration in the Azolla plant may be due to active components in this plant, which can inhibit free radical activity within the animal's body as one of the most important natural antioxidants [174]. On the other hand, dietary supplementation of docosahexaenoic acid-rich microalgae (0.25–1.0%) significantly increased egg eicosapentaenoic acid and docosahexaenoic acid concentrations while decreasing egg n-6 : n-3 ratio, making eggs more nutritionally valuable [92].

## 6. Conclusions

The egg industry faces several challenges, the most pressing of which is providing healthy and high-quality products to consumers. In recent years, market demand for functional foods has increased. Modifications and alterations in laying hen diet composition greatly impact egg nutritional composition and quality preservation. These dietary changes can potentially improve fatty acid and pigment concentrations while decreasing bad cholesterol levels in eggs. According to the scientific literature, designed-enriched egg products contain higher levels of desirable nutrients, such as omega-3 fatty acids, and their consumption can benefit human health. On the other hand, consumers have expressed a strong desire for animal welfare to be considered in poultry production, including table eggs. Because of societal pressures, several table-egg farms have shifted from conventional to enriched cages. They also changed from raising their birds in cages to noncage production systems, supporting birds' behavior and welfare. These changes can have unintended consequences in the future, such as disease exposure. Hen laying persistency will be routinely extended shortly to improve sustainability and profitability and reduce egg production's impact on the environment. Keeping laying hens productive for 100 weeks can save production costs and breeding time, as well as improve hen welfare. However, scientists and breeders are facing a great challenge in optimizing genetics, production systems, and nutrition to ensure hen health and high product quality. The present review discussed several practical strategies to increase layers' egg production and welfare and produce healthy/safe eggs. These practical strategies can potentially be used in layer farms to sustain the egg industry. One of these strategies is to use plant/herbal substances as dietary supplementation in poultry diets on a large scale. They are recommended in poultry farms as natural feed additives and alternatives to any artificial or synthetic chemical material, such as antibiotics. These natural substances can be added to the layer diet separately or in combination to improve hens' physiological, productive, reproductive, and immunological performance, which has the greatest impact on bird welfare and quantitative and qualitative productivity.

## Figures and Tables

**Table 1:** Practical breeding, husbandry, and nutritional strategies to improve layer egg production and quality.

The strategy	Studied traits	Species	Main results	Reference
Marker-assisted breeding selection	Egg production candidate genes	Laying chickens	Promising genes and SNP markers could be used to improve egg production and quality using marker-assisted breeding selection.	[19–21]

Transgenic technology	Egg products, human immunity	Laying chickens	Possibility of using novel biotechnology techniques to genetically modify hens to produce eggs with medical benefits such as insulin and antibodies.	[8, 22, 23]
			In addition, using eggs as a source of immunogens that enhance the immune system of humans.	

Housing system enrichment	Birds' behavior and welfare	Laying chickens	Using enriched cages, such as providing additional feeders, and free-range housing systems can improve laying hen behavior and welfare.	[24–26]
	Egg production and quality		Hens reared in enriched cages showed higher production performance and egg quality than those reared in the conventional cages and aviary system.	[27]

Environmental-enriched cages	Birds' behavioral activities and density	Laying chickens	Environmental enriched cages encourage the daily activities of birds and provide more opportunities for better space utilization. Sufficient space in cages and aviaries is preferable for birds to express natural behaviors, such as perching, nesting, dust bathing, and walking.	[28–30]

Egg enrichment	Egg production and quality	Laying chickens	Feeding laying hens high PUFA with a lower linoleic acid to *α*-linolenic acid ratio in the diet can result in PUFA-enriched eggs.	[31]
			Adding a mixture of 25% marigold flower meal (*Tagetes erectus*) and 75% spinach (*Spinacia oleracea*) improved egg yolk color.	[32]
			Egg lycopene enrichment can be performed by supplementing feed with tomato powder (5–10 g/kg diet). This supplementation increased egg production and improved egg yolk color.	[33]

Dietary supplementation	Egg production and quality	Breeder hens	Supplementing the diet with quercetin and vitamin E increased the laying rate (84.5%), enhanced immunity response (IgM and IgA concentrations), and improved the yolk weight, yolk height, and Haugh unit in the aging breeder hens compared with those fed a basal diet.	[34]
		Laying chickens	Dry thyme leaves (2%) and thyme extracts are advised to be adding in laying farms to improve laying hens' physiological and productive performance, as well as egg quality by increasing n-3 FA concentrations and decreasing bad yolk cholesterol.	[35, 36]
	Egg quality and fecal microbiota	Laying chickens	Supplementing extruded flaxseed meal (up to 20%) in laying hen diets increased *α*-linolenic acid and docosahexaenoic acid deposition in egg yolk. Increasing *α*-linolenic acid positively influenced egg quality and fecal microbiota.	[37, 38]
	Egg weight and quality	Laying Japanese quails	Oral administration of 100 to 150 *μ*l/kg body weight of ginger essential oil to laying quails increases egg weight and lowers egg cholesterol.	[39]

## Data Availability

The data used to support the findings of this study can be obtained from the corresponding author upon request.
